# Soluble fibrinogen like protein 2 (sFGL2), the novel effector molecule for immunoregulation

**DOI:** 10.18632/oncotarget.12533

**Published:** 2016-10-08

**Authors:** Xin-guang Liu, Yu Liu, Feng Chen

**Affiliations:** ^1^ Department of Hematology, Qilu Hospital, Shandong University, Jinan, P. R. China; ^2^ School of Chemistry and Pharmaceutical Engineering, Qilu University of Technology, Jinan, P. R. China; ^3^ Capital Medical University Cancer Center, Beijing Shijitan Hospital, Beijing Key Laboratory for Therapeutic Cancer Vaccines, Beijing, China

**Keywords:** soluble fibrinogen-like protein 2, immunoregulation, transplantation, hepatitis, autoimmunity

## Abstract

Soluble fibrinogen-like protein 2 (sFGL2) is the soluble form of fibrinogen-like protein 2 belonging to the fibrinogen-related protein superfamily. It is now well characterized that sFGL2 is mainly secreted by regulatory T cell (Treg) populations, and exerts potently immunosuppressive activities. By repressing not only the differentiation and proliferation of T cells but also the maturation of dendritic cells (DCs), sFGL2 acts largely as an immunosuppressant. Moreover, sFGL2 also induces apoptosis of B cells, tubular epithelial cells (TECs), sinusoidal endothelial cells (SECs), and hepatocytes. This mini-review focuses primarily on the recent literature with respect to the signaling mechanism of sFGL2 in immunomodulation, and discusses the clinical implications of sFGL2 in transplantation, hepatitis, autoimmunity, and tumors.

## INTRODUCTION

Fibrinogen-like protein 2 (FGL2), also known as fibroleukin, is identified as a member of the fibrinogen-related protein superfamily (fibrinogen-related domain, FRED) because of its homology with fibrinogen β and γ chains [1]. Members of this protein family also include fibrinogen, tenascin, ficolin, angiopoietin, and fibronectin, which are capable of adjusting the host's immune response [2–6]. The fgl2 gene was originally cloned from cytotoxic T lymphocytes (CLTs), and the encoded glycoprotein shared a 36% homology to the β and γ chains of fibrinogen, and a 40% homology to the FRED of tenascin [7, 8]. It is well known that FGL2 has two structurally different forms: the membrane bound FGL2 (mFGL2) and the soluble FGL2 (sFGL2). mFGL2, a 70 kDa type II transmembrane glycoprotein protein expressed on the surface of macrophages or endothelial cells, is a direct prothrombinase with serine protease activity which can cleave prothrombin into thrombin through a noncanonical pathway, thus exerting procoagulant activity in immune-associated coagulation [9–11]. By contrast, sFGL2 has a 50 kDa weight and is highly expressed by CD4+CD25+ regulatory T cells (Tregs) [12–15]. Moreover, sFGL2 expressed by other Treg populations, including CD8+CD45RClow T cells [16], CD8αα+ suppressive intraepithelial lymphocytes (IELs) [17], and CD3+CD4-CD8- double negative T cells (DNTs) [12, 18], also accounts for a small proportion of sFGL2 origin. Therefore, sFGL2 might be a common effector molecule of many classes of Tregs. Functionally distinct from mFGL2, sFGL2 possesses abilities in immunomodulation and contradictory properties in tissue injuries [15, 19, 20]. Recently, a growing body of evidence indicated that FLG2 was involved in the pathogenesis of a variety of diseases such as pregnancy failure [21], tumor growth [22], viral infection [23–25], allograft rejection [26], and autoimmune disorders [12, 27]. As the physiological function and the pathogenetic roles of mFGL2 have been well elucidated in some other papers [20, 28, 29], this mini-review will focus on recent progress about the signaling mechanism of sFGL2 in immunomodulation. Furthermore, the characteristics and therapeutic potential of sFGL2 in transplantation rejection, viral hepatitis, autoimmunity, and tumors will also be discussed.

## GENE ENCODING, PROTEIN STRUCTURE, AND EXPRESSION REGULATION OF SFGL2

**Figure 1:**
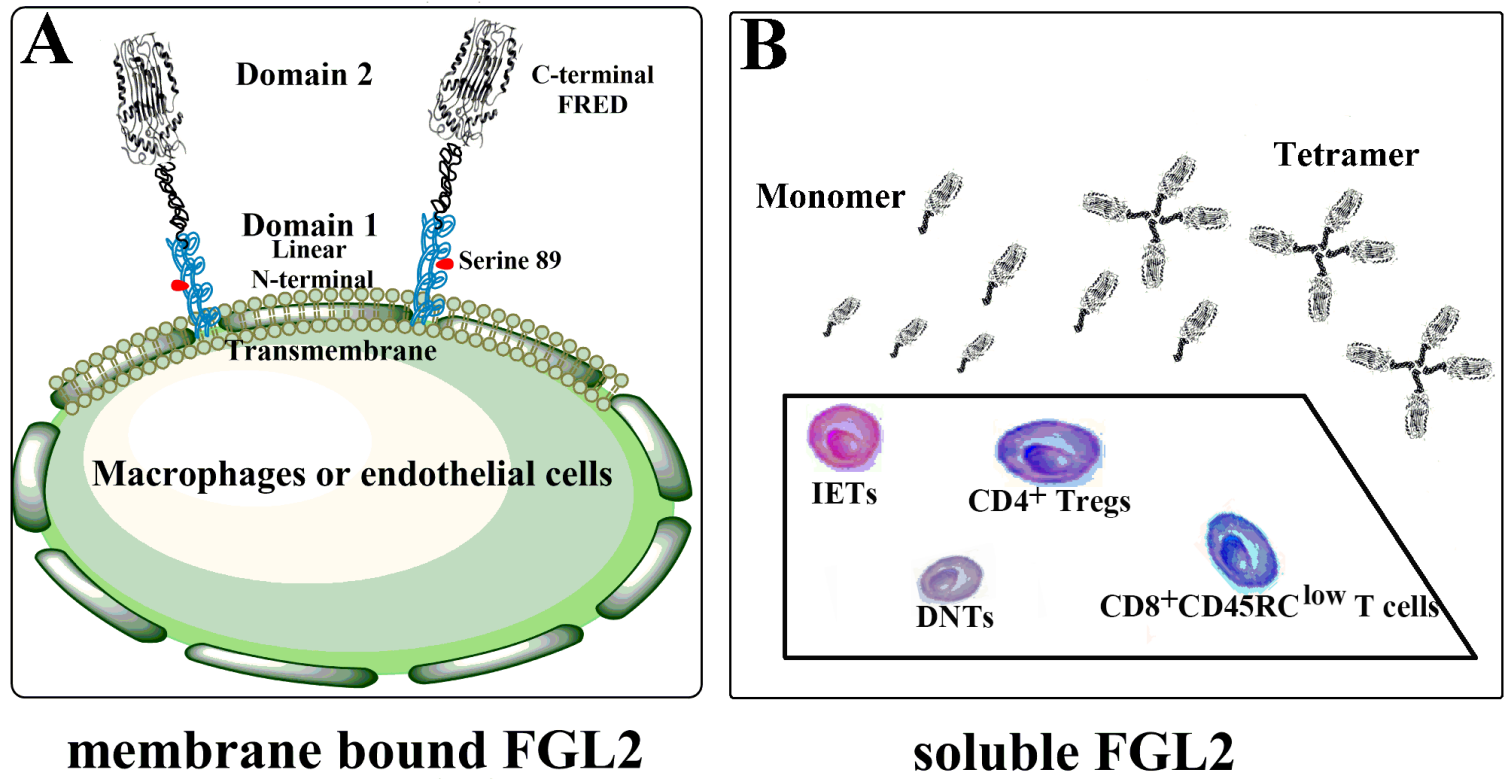
Schematic view of the two forms of FGL2 **A.** Membrane bound FGL2 (mFGL2) is a type II transmembrance glycoprotein expressed by macrophages or endothelial cells. The N-terminal linear coiled-coil domain (Serine 89) of mFGL2 is responsible for its prothrombinase activity, while function of C-terminal FRED domain remains unclarified. **B.** Soluble FGL2 (sFGL2) lacking the N-terminal hydrophobic sequence is mainly secreted by CD4+CD25+Tregs. sFGL2 expressed by CD8+CD45RClow T cells, CD8αα+ suppressive intraepithelial lymphocytes (IELs), and CD3+CD4-CD8- double negative T cells (DNTs) also accounts for a small proportion of sFGL2 origin. sFGL2 in its natural state exists as an oligomer consisting of 4 monomers.

The flg2 gene, localized to the proximal region of chromosome 7q11.23 in humans and 5 in mice, is composed of two exons that are separated by one intron [11]. The longest open reading frame (ORF) of FGL2 encodes a protein of 439 amino acids in humans and 432 amino acids in mice, respectively. Transcription of human flg2 gene can produce 4 different mRNAs, 3 alternatively spliced variants, and 1 unspliced form [30]. The detailed cleavage manner leading to the function divergence between mFLG2 and sFGL2 remains unclear. Amino acid sequence analysis of FGL2 revealed an N-terminal hydrophobic motif which had a linear conformation, and a 229-amino-acid-long carboxyl-terminal domain known as FRED (Figure 1A) [9, 31, 32]. The serine 89 residue of the N-terminal domain was shown to be accounted for the procoagulant activity of mFGL2 [10], while FRED was reported to be critical for sFGL2-mediated immunoregulation [8]. A stretch of hydrophobic amino acids at N-terminus of FGL2 served as signal peptide for sFGL2 secretion, but how sFGL2 was cleaved and secreted remained unknown [11]. Liu and colleagues recently showed that glycosylations of the amino acids at positions of 172, 228, 256, and 329 were responsible for maintaining the solubility of sFGL2 [15]. sFGL2 in its natural state existed as an oligomer consisting of 4 monomers (Figure 1B) [33, 34]. Through inter-chain disulfide bond of cysteinesat amino acid positions 94, 97, 184, and 187 at the central and C terminal region, sFGl2 monomers are assembled into dimers, and next dimers are further assembled into tetramers by inter-chain disulfide bonds with two additional cysteine pairs (Figure 1) [15]. Interestingly, monomeric sFLG2 has been found to exhibit greater immunosuppressive activity than native oligomer sFGL2 [15]. Similar to the structure in the D domain of fibrinogen, two pairs of cysteins located in the FRED of sFGL2, Cys206-Cys235 and Cys364-Cys377, could form intra-chain loops which might affect the biological activity of sFGL2 [15].

In contrast with mFGL2 which is expressed by endothelial cells, epithelial cells, macrophages, and dendritic cells (DCs) [35], sFGL2 has been found to be secreted by CD4+CD25+ Tregs, CD8+CD45RClow T cells, IELs, and DNTs, but not by T helper (Th) cells and B cells [7, 12, 16, 17, 33, 36]. Various transcription factors, such as Ets, TCF1, CEBP, AP1, Ikaros or SP1, can bind FGL2 promoter through cis element consensus sequences, contributing to the differential transcription and expression pattern of FGL2 based on different cell types [11]. More specifically, members of the Ets transcription factor family are crucial for FGL2 expression in vascular endothelium, whereas Ikaros along with TCFI account for FGL2 transcription in lymphocytes [37]. Additionally, CEBP/a is responsible for the constitutive expression of FGL2 in hepatocytes, while recruitment of CEBP/b contributes to the induction of this gene in macrophages [11, 38].

## ROLE OF SFGL2 IN IMMUNO-REGULATION

### sFGL2 and antigen-presenting cells

It has been well established that sFGL2 lacks procoagulant activity of mFGL2, but rather functions as a multimodality regulatory of immunosuppression [8]. sFGL2 cannot bind to fibrinogen receptors such as Mac-1, ICAM-1 or Toll-like receptor 4 (TLR4). Up to now, two receptors, Fcγ receptor (FcγR) IIB and FcγRIII, have been identified as the mediator of sFGL2 function [39]. FcγRIIB has an immunoreceptor tyrosine-based inhibition motif (ITIM) in its intracytoplasmic domain [40], and is the only FcγR that has an inhibitory function [41]. On the contrary, FcγRIII contains an immunoreceptor tyrosine-based inhibition motif (ITAM) that mediates the activating signaling [42]. After binding to FcγRs, sFGL2 has distinct biological effects on diverse cell types, which might be due to different expression ratio of FcγRIIB to FcγRIII on cellular surface, or different affinities of sFGL2 to that two kinds of FcγRs [43].

Antigen-presenting cells (APCs), including DCs, monocytes/macrophages, and B cells, play important roles not only in innate immunity but also in adaptive immune responses. By binding to FcγRIIB and FcγRIII, sFGL2 can adjust the antigen presentation ability of APCs. Target deletion of FGL2 in mice resulted in increased number of Ab-producing B cells and DCs in spleen [12]. Moreover, anti-viral B cell responses following Lymphocytic Choriomeningitis Virus WE infection were remarkably enhanced in fgl2-/- mice, which was beneficial to virus elimination [44]. Following lipopolysaccharide (LPS) stimulation, elevated levels of CD80 and major histocompatibility complex (MHC) II as well as decreased ratio of apoptosis were observed on DCs from fgl2-/- mice [12].

On the contrary, exogenous sFGL2 could lead to reduced expression of CD80 and MHC II on bone marrow (BM)-derived DCs, while levels of MHC I and CD86 on DCs were not challenged [8]. Given the crucial roles of co-stimulatory molecules played in the initiation of immune response by T and B cells, it is not surprising that modulation of these molecules on DCs by sFGL2 would have profound effect on T cell immunity of the adaptive immune system. Studies by Chan revealed that sFGL2 could prevent the maturation of DCs and reduce their capacity to stimulate T cell proliferation [8]. FcγRIIB and FcγRIII are also expressed by monocytes/macrophages, but in what way they are modulated by sFGL2 still needs further investigation.

### sFGL2 and T cells

It has been widely recognized that sFGL2 acts as an important effector molecule of CD4+CD25+ Tregs in their development and function. Previous study has shown that CD4+CD25+ Tregs were more abundant in fgl2-/- mice, but their ability to suppress effector CD4+ T cell proliferation was significantly impaired. In contrast to the blockade of IL-10, TGF-β or CTLA-4 which was ineffective or weak in CD4+CD25+ Treg activity inhibition, administration of sFGL2 neutralizing antibody could abolish the suppressive activity of CD4+CD25+ Tregs in vitro in a dose dependent manner [12]. In addition, Th cells from fgl2-/- mice were polarized toward a Th1 phenotype [12]. FGL2 and PD-1 have been identified as the most upregulated genes in perinatally generated CD4+CD25+ Tregs which play critical roles in maintaining self-tolerance during life [45]. More recently, Joller et al. revealed that sFGL2 was indispensable for the ability of the novel identified TIGIT+CD4+CD25+ Treg cell subset to suppress Th1 and Th17 cell response [46]. Taken together, sFGL2-mediated immunoregulation might be crucial for the maintenance of Th cell homestasis.

Aside from CD4+CD25+ Tregs, other Treg cell subsets were also capable of producing sFGL2. Li and colleagues found that FGL2 expressed by tolerogenic CD8+CD45RClow Tregs was an important mediator of CD8+ Treg suppression [16]. An obvious expression of FGL2 in DNTs has been observed, and FLG2 deprivation in DNTs was associated with the loss of suppressive activity [12, 18]. In addition, Denning demonstrated that FGL2 mRNA was highly expressed in TCRαβ+CD8αα intestinal IELs [17], a CD8+ T cell subtype critical for the establishment of normal mucosal tolerance [47]. But the precise effect of sFGL2 expressed by TCRαβ+CD8αα IELs on mucosal immunity has not been clarified yet. Collectively, these data suggest that sFGL2 is a common immunosuppressive mediator of many Treg subsets (Table 1).

**Table 1:** sFGL2-expressing Tregs

	CD4+ Tregs	CD8+ Tregs	IELs	DNTs
Immunophenotypic identification	CD4+CD25highFoxp3+	CD45RClow	CD8αα+	CD3+CD4-CD8-
Suppressive, pro-apoptotic, and cytolysis molecules	CTLA-4, LAG3, LAP, TIGIT, IL-10, TGF-β, IL-35, PD-1, CD95, GITR, galectin 1, granzymes	CTLA-4, IL-10, TGF-β, IDO, FasL, perforin	TGF-β3, LAG3, FasL, CD69, granzymes, NK-like receptors	CTLA4, FasL, perforin,
Origin	Thymus (nTreg) or periphery (iTreg)	Thymus	Intestinal epithelium	Thymus or periphery
Mechanisms	Attenuation of DC function, inhibition of Th1 and Th17 development, anti-inflammatroy apoptosis induction, Breg induction	Perforin-mediated cytolysis, FasL-induced apoptosis, induction of CD4+ Tregs, inhibitory cytokine-mediated suppression	Fas-and perforin-mediated cytotoxicity, inhibitory cytokine-mediated suppression	Perforin-mediated cytolysis, FasL-induced apoptosis, Attenuation of DC function
References	[12–14, 70, 106]	[16, 107]	[17, 108, 109]	[12, 107]

IELs, intraepithelial lymphocytes; DNTs, double negative T cells; CTLA-4, cytotoxic T lymphocyte-associated antigen 4; LAG3, lymphocyte activation gene 3; LAP, latency-associated peptide; TIGIT, T-cell Ig and ITIM domain; IL, interleukin; TGF, transforming growth factor; PD-1, programmed cell death 1; GITR, glucocorticoid-induced TNF-receptor-related protein; IDO, indoleamine 2,3-dioxygenase; FasL, Fas ligand; nTregs, natural Treg; iTreg, induced Treg.

### sFGL2 and regulatory B cells

B cells play important roles in innate and adaptive immunity. Over the past decade, a proportion of B cells with suppressive function have gained substantial attention. That B cell subset is collectively known as regulatory B cells (Bregs) which act as modulators of the immune response against pathogens and autoantigens. Bregs exert their regulatory effect through producing anti-inflammatory cytokines, inducing the apoptosis of effector T cells, and promoting the differentiation of CD4+CD25+Foxp3+ Tregs [48, 49]. More recently, Bezie and co-workers demonstrated that adoptive transfer of splenocytes from FGL2-overexpressed rats into animals that were transplanted with cardiac allografts could prevent acute and chronic rejection. FGL2-overexpression in donor rats favored the proliferation of CD45 RA+ Bregs in spleen, and these Bregs were the main cells responsible for the induction of allograft tolerance [50].

### Roles of sFGL2 in apoptosis

sFGL2 also functions as a proapoptotic effector molecule besides its well identified immunosuppressive activity. Through binding to the inhibitory FcγRIIb, sFGL2 could promote cellular apoptosis of sinusoidal endothelial cells (SEC) and hepatocytes, leading to hepatic reperfusion injury [51]. In a porcine kidney auto-transplantation model, peripheral and local sFGL2 levels during the recovery of renal ischemia reperfusion injury were significantly elevated [51]. Additionally, in renal allograft recipients with acute rejection (AR), serum levels of sFGL2 were shown to be increased to an extent dependent on pathological severity [52]. Further study by the same group revealed that in renal allograft patients with AR, elevated sFGL2 could induce tubular epithelial cell (TEC) apoptosis [53]. sFGL2 was also involved in the induction of B cell apoptosis. Shalev et al. reported that FcγRIIb positive A20 B cells treated with recombinant sFGL2 would undergo obvious apoptosis process [12]. B cells are composed of B1 cells, B2 cells, and Bregs [54]. These B-cell subsets play distinct and non-redundant roles in immunoregulation. Nonetheless, the impact of sFGL2 on these different B-cell subsets still awaits further exploration.

### sFGL2-related signaling pathways

**Figure 2:**
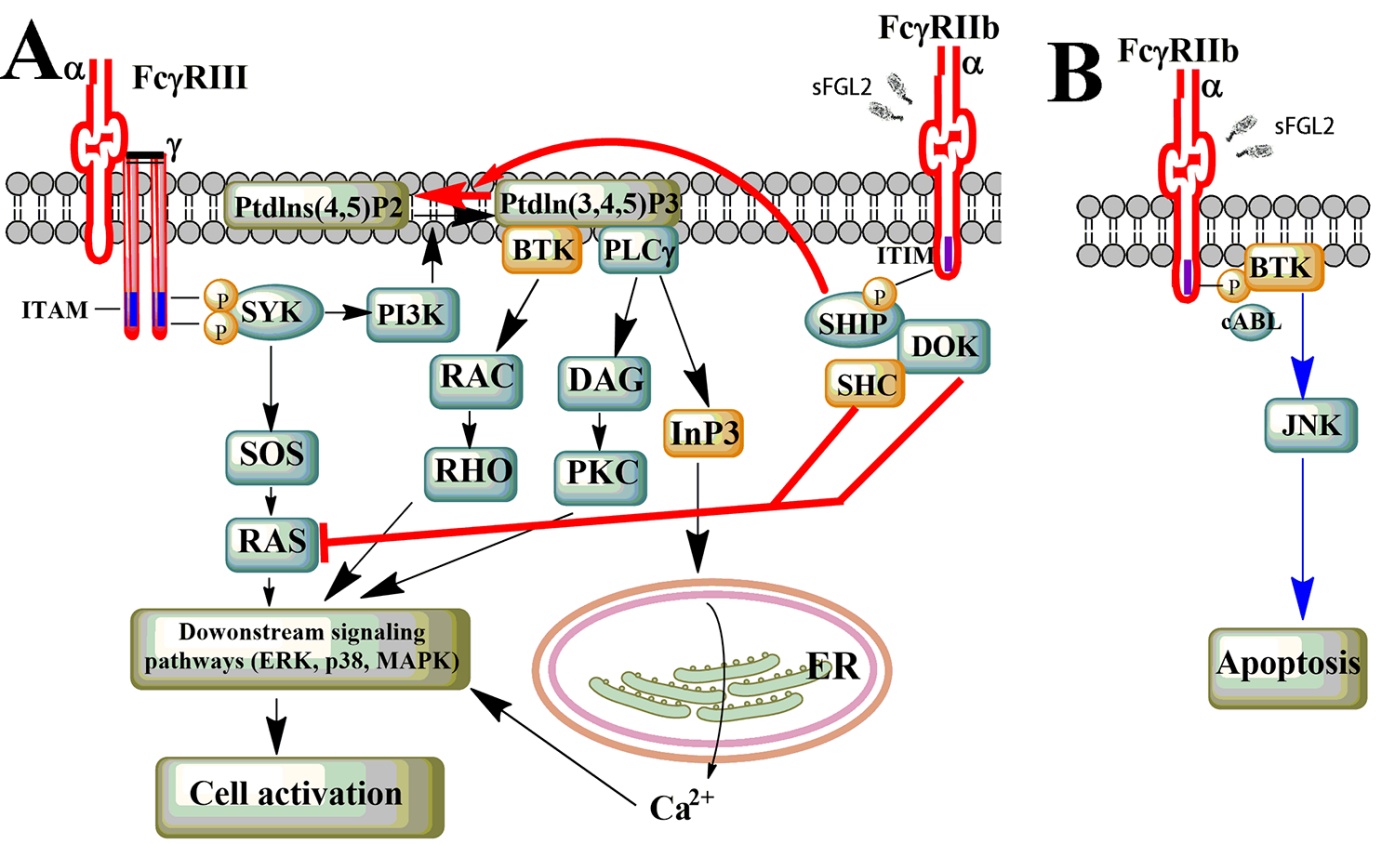
Potential sFGL2-related signaling pathway **A.** ITAM/ITIM-dependent signaling pathway. Upon FcγRIII crosslinking, LYN phosphorylates ITAM in the cytoplasmic domain of FcγR γ-chain, this creates SRC homology 2 (SH2) docking sites for SYK, which subsequently activates PI3K and SOS, et al. Generation of Ptdlns (3,4,5)P3 by PI3K recruits BTK and PLCγ, which further leads to activation of downstream kinases and the release of calcium from ER. Binding of sFGL2 to FcγRIIb leads to phosphorylation of the ITIM in the cytoplasmic tail of FcγRIIb by LYN. This results in hydrolysis of Ptdlns (3,4,5)P3 into Ptdlns (3,4,5)P2 and inhibition of RAS, thus attenuating the activating-FcγR-mediated cell activation. **B.** Triggering of FcγRIIb can lead to cell apoptosis through ITIM- and SHIP-independent signaling pathways which involve the cABL kinase family, BTK, and JNK.

It has been well established that sFGL2 exerts multiple effects by interacting with its receptors, FcγRIIb and FcγRIII. Nevertheless, the downstream signaling pathways after ligand-receptor binding still remain unilluminated. According to previously identified signaling pathways that can be triggered by FcγR crosslinking, several pathways are proposed to be related to the function of sFGL2 (Figure 2).

### ITAM/ITIM-dependent signaling pathway

FcγRIII is one of the activating FcγRs composed of a ligand-binding α-chain and a single-transducing γ-chain dimer which carries an immunoreceptor tyrosine based activating motif (ITAM). While FcγRIIb is a single α-chain inhibitor receptor which contains an immunoreceptor tyrosine based inhibitory motif (ITIM) in its cytoplasmic domain [41]. To date, our existing knowledge about the FcγR-related pathways are primary derived from crosslinking of FcγRs by immune complexes (ICs) [41, 55]. FcγRIII crosslinking by ICs induces tyrosine phosphorylation of the ITAM in the receptor-associated adaptor molecules by kinases of the SRC family. This creates SRC homology 2 (SH2) docking sites for SYK-family kinases, which subsequently activates a variety of downstream signal-transduction molecules such as phosphoinositide 3-kinase (PI3K), multimolecular adaptor complexes, son of sevenless homologue (SOS), or the linker for activation of T cells (LAT). The generation of phosphatidylinositol-3,4,5-trisphosphate (Ptdlns(3,4,5)P3) by PI3K can recruit Bruton's tyrosine kinase (BTK) and phospholipase Cγ (PLCγ), which leads to the release of calcium from the endoplasmic reticulum (ER). Intracellular calcium level increase can induce a number of downstream signaling events and trigger multiple cellular activities [56, 57]. Apart from calcium-dependent pathway, the RAS-RAF-MAPK-pathway can be activated through SOS bound to Grb2 that is recruited on phosphorylation of SHC, and is of critical importance for cell activation following crosslinking of the activating FcγRs.

Crosslinking of the inhibitory FcγRIIb by ICs can dampen the activating pathways by interfering with the generation of key intermediates such as Ptdlns(3,4,5)P3. Phosphorylation of the ITIM motif in the cytoplasmic portion of FcγRIIb by LYN leads to the recruitment of the SH2-domain-containing inositol-5-phosphatase (SHIP) and the hydrolysis of Ptdlns(3,4,5)P3 into Ptdlns(4,5)P2, which ultimately inhibits recruitment of pleckstrin homology (PH)-domain containing proteins such as BTK and PLCγ, and thereby attenuating ITAM-signaling-mediated calcium release and downstream effector function [58]. Moreover, the RAS-RAF-MAPK signaling pathway could also be inhibited by recruitment of SHC tyrosine-phosphorylated SHIP.

### ITAM/ITIM-independent signaling pathway

It was reported a decade ago that isolated triggering of FcγRIIb could induce B cell apoptosis [59]. Similarly, homo-oligomerization FcγRIIb would result in elevated levels of B-cell death, and it was demonstrated later that a signaling pathway involving the cABL kinase family, BTK and JUN N-terminal kinase (JNK), but independent of SHIP and ITIM, was responsible for this phenotype [60, 61]. Furthermore, co-engagement of FcγRIIb and BCR in SHIP-/- B cells also led to apoptosis [59, 62]. Nevertheless, what we know about the impact of sFGL2 on ITIM-independent pathway is relatively rare, and further investigation is needed to verify the relationship between the signaling pathway and sFGL2-mediated apoptosis in B cells, TECs, SECs, or hepatocytes.

## CLINICAL SIGNIFICANCE OF SFGL2

### sFGL2 in transplantation

Transplantation has been emerged as a viable therapeutic modality for the management of end-stage renal, cardiac, and liver failure. However, rejection still remains the biggest impediment to long-term survival of both allograft and recipients [63]. In line with that, seeking of novel immunosuppressive agents has become a hot issue in the field of transplant immunology [64, 65]. Numerous studies have shown that CD4+CD25+ Tregs played critical roles in the induction and maintenance of transplant tolerance [66–69]. Molecular mechanisms of immune suppression by CD4+CD25+ Tregs include modulation of cytokine microenvironment, metabolic disruption of the target cells, alteration of DC activating capacity and cytolysis [14, 70, 71]. As an effector molecule of CD4+CD25+ Tregs, sFGL2 was also found to be involved in the induction of transplant tolerance. In tolerant cardiac and liver allografts, expression of sFGL2 as well as other tolerogenic set of genes such as forkhead box P3 (Foxp3), cytotoxic T lymphocyte-associated protein 4 (CTLA4), and killer cell lectin-like receptor G1 (Klrg1) was significantly elevated [72]. Numbers and immunosuppressive activity of CD4+ Tregs were significantly elevated in cardiac allografts of fgl2 transgenic mice, whereas proliferative ability of effector T cells to alloantigen stimulation was remarkably decreased [73]. Rapamycin-induced tolerance in C3H/HeJ mice transplanted with MHC-mismatched hearts from BALB/cJ mice was associated with elevated plasma levels of sFGL2, and FoxP3/FGL2 dual positive CD4+CD25+ Tregs were remarkably increased in tolerant allografts. It is notable that treatment with anti-FGL2 antibody could reverse the tolerizing effects of rapamycin in vitro and in vivo, indicating a key role of sFGL2 in transplantation tolerance induction [74]. Bezie et al. confirmed that sFGL2 expression was significantly upregulated in the graft and splenic CD8+CD45RClow regulatory T cells of cardiac transplant rat at mRNA and protein level, and sFGL2 could induce long-term transplantation tolerance which was active and transferable by splenocytes [75]. The same group further observed in cardiac allotransplantation rats that FGL2 over-expression in vivo through virus-mediated gene transfer resulted in inhibition of cardiac allograft rejection. The established allograft tolerance was transferable and Bregs induced by FGL2 over-expression were the main cells responsible for this effect [50]. As mentioned in a previous section, serum levels of sFGL2 were increased among renal allograft recipients with biopsy-proven acute rejection versus those with stable allograft or healthy controls. Moreover, serum sFGL2 levels were remarkably elevated in patients with antibody-mediated than T-cell-mediated acute rejection episodes, and sFGL2 levels in patients with grade II T-cell-mediated rejection were significantly higher compared with that in patients with grade I T-cell-mediated rejection, indicating that sFGL2 expression in renal transplant patients with rejection was dependent on the pathological type and severity of the response [52]. Zhu and colleagues further discussed in their paper that decreased serum levels of sFGL2 in the stable group might be due to attraction of Tregs to the graft with sFGL2 secretion, reducing the content in the peripheral blood [43].

**Figure 3:**
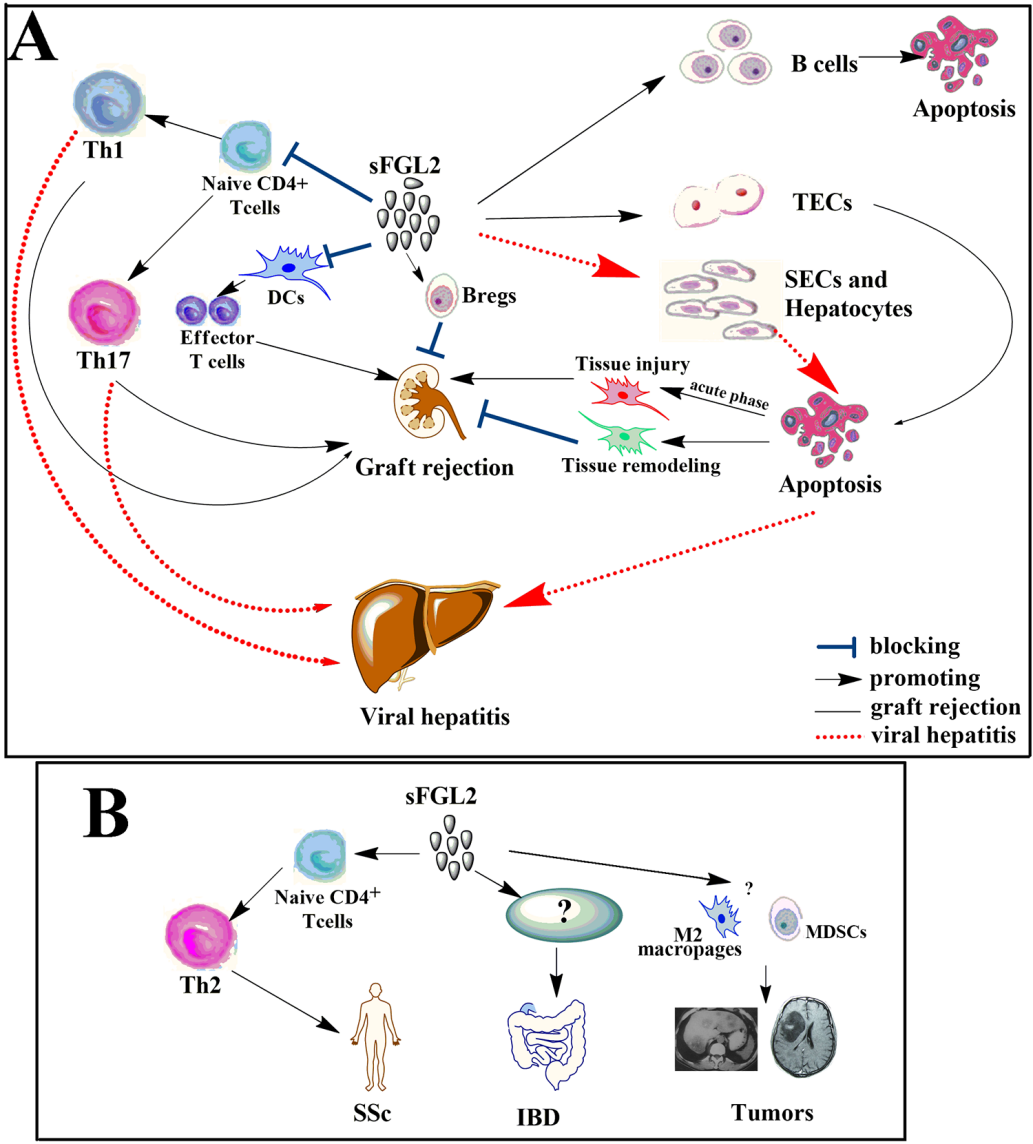
sFGL2 in transplantation, viral hepatitis and autoimmunity **A.** By inducing apoptosis of TECs, repressing the maturation of DCs, promoting the differentiation and proliferation of Bregs, and inhibiting differentiation of Th1 as well as Th17 cells, sFGL2 exerts protective roles in the recovery phase after renal transplantation. However, apoptosis of TECs during the acute phase leads to kidney injury. In viral hepatitis, sFGL2 not only induces apoptosis of SECs and hepatocytes but also impairs anti-viral immunity by inhibiting differentiation of Th1 and Th17 cells, thus contributing to liver injury. **B.** sFGL2 plays a pathogenetic role in the development of SSc by promoting Th2 polarization. While roles of sFGL2 upregulation in IBD still need further investigation. sFGL2 might promote tumor progression by increasing the frequencies of myeloid-derived suppressor cells (MDSCs), M2 macrophages, upregulating the co-inhibitory receptor TIGIT expression, and inhibiting the anti-tumor effect of CD8+ T cells.

Apart from immune tolerance induction, sFGL2 could promote renal graft survival by inducing apoptosis of TECs. Data emerging from the literature suggested that TEC apoptosis was engaged in the pathogenetic process of kidney allograft rejection. Based on the results of experimental models of acute renal failure (ARF) in vivo, TEC apoptosis could be divided into two distinct phases. The first phase occurred shortly after the acute ischemic or nephrotoxic insult, which probably contributed to tubular cell loss and the tubular dysfunction associated with ARF. Whereas the second phase of apoptosis took place many days later during the recovery phase of ARF, which could provide some beneficial effects on the remodeling of injured tubules (Figure 3A) [76–78]. With regard to renal allograft, we might deduce that impact of sFGL2-induced TEC apoptosis was related to the stages after kidney transplant.

Infections are a major determinant of the outcome of organ transplantation and remain a significant cause of mortality among the recipients [79, 80]. It is well known that current steroids or steroid-free immunosuppression schemes after organ transplantation could increase susceptibility to opportunistic infections [80–82]. Consequently, novel immunosuppressants are urgently needed to overcome or minimize adverse effects of immunosuppressive agents currently available. Liu et al. observed that sFGL2 injection into C57BL/6J mice could significantly prolong survival of skin allograft from 7.8 ± 1.99 d to 15 ± 2.56 d [39]. This finding indicated that sFGL2 was capable of inducing immune tolerance independent of prolonged immunosuppression, thus attenuating the risk of infection, secondary neoplasia, or cardiovascular disease. It was notable that monomeric sFGL2 possessed greater immunosuppressive activity than native oligomer sFGL2, so monomeric sFGL2 might be more potential for clinical use than native sFGL2 in term of its stronger immunosuppressive potency, higher permeability, and less antigenicity because of its lower molecular weight [15, 35].

### sFGL2 in hepatitis

sFGL2 secreted by Tregs could suppress T cell immune responses to viral infections including hepatitis C virus (HCV) and HBV, thus might contribute to the development of viral hepatitis (Figure 3). Animal studies have demonstrated that plasma levels of sFGL2 in BALB/cJ mice were significantly increased after murine hepatitis virus 3 (MHV-3) infection, and treatment with anti-FGL2 antibody or antisense plasmid complementary to the exon 1 of FGL2 gene could protect susceptible BALB/cJ mice against MHV-3-induced fulminant viral hepatitis (FH) and mortality [13, 83]. In patients with biopsy-proven HCV hepatitis, plasma levels of sFGL2 were considerably increased and correlated positively with HCV titers as well as degree of inflammation in the liver. Following an effective anti-viral therapy, levels of sFGL2 decreased significantly [23]. Compared with patients without cirrhosis or patients with inactive end stage alcoholic cirrhosis, HCV-patients with cirrhosis showed higher levels of sFGL2 [23]. Likewise, plasma levels of sFGL2 in patients with non-alcoholic steatohepatitis (NASH) and borderline NASH were significantly higher than that in healthy controls [84].

### sFGL2 in autoimmune diseases

Systemic sclerosis (SSc) is generally identified as a Th2-dominant autoimmune disease [85]. Yanaba and co-workers recently reported that serum sFGL2 levels were increased remarkably in patients with SSc compared with healthy controls or systemic erythematosus lupus (SLE) [86]. By promoting Th2 polarization and inducing vascular endothelial damage in ischemia reperfusion injury, sFGL2 was thought to play a pathogenetic role in the development of SSc (Figure 3B).

Inflammatory bowel disease (IBD) is another autoimmune disorder which has been reported to have aberrant sFGL2 expression. The work of Dong revealed that mRNA and protein expression levels of FGL2 in peripheral blood mononuclear cells (PBMCs), as well as concentrations of sFGL2 in plasma were remarkably upregulated in both ulcerative colitis (UC) and Chohn's disease (CD) patients compared with healthy controls. Moreover, UC and CD patients with active disease showed higher sFGL2 levels than those with inactive disease, and levels of sFGL2 correlated positively with disease activity indices, C-reactive protein (CRP), and erythrocyte sedimentation rate (ESR) [87]. Upregulation of sFGL2 in active UC and CD patients might be attributed to the insufficient compensation secreted by Tregs which failed to control the chronic inflammation.

It has been widely believed that aberrant Th profile is involved in the pathogenesis of a variety of autoimmune diseases. For example, primary immune thrombocytopenia (ITP) often exhibits a Th1 dominant profile together with elevated Th17 expression [88, 89]. In SLE patients, the role of Th1/Th2 balance in the development of SLE remains controversial, as both decreased and unchanged Th1 profile in peripheral blood has been shown previously [90, 91]. However, Th17 subset in peripheral blood has been consistently reported to be increased in patients with SLE, and the elevated peripheral Th17 frequency correlated positively with the disease activity [92]. Furthermore, the pathogenetic role of elevated Th17 cells has also been established in several other autoimmune disorders, such as rheumatoid arthritis (RA) [93, 94] and psoriasis [95, 96]. The critical role of sFGL2 in limiting Th1 and Th17 responses warrants further investigation in these autoimmune diseases.

### sFGL2 in tumors

Tumors often constitute highly immunosuppressive microenvironments in which enhanced suppressive capacity of Tregs and impaired function of effector T cells coexist [97, 98]. Based on the documented role of sFGL2 as a Treg effector molecule, it is reasonable to speculate that sFGL2 might play a role in inhibiting anti-tumor immune responses. Indeed, Birkhäuser et al observed significantly elevated expression of FGL2 together with remarkably upregulated expression of CCL1, CXCL9, and HMGB1 in a murine renal carcinoma model which was not responsive to a DC-based vaccine therapy [99], and the altered gene expression profile was thought to contribute to evasion of immune surveillance in tumors [20]. More recently, Yan and colleagues reported that FLG2 contributed to the progression of glioblastoma multiforme (GBM) through inducing multiple immunosuppression mechanisms [100]. Compared with low-grade gliomas, GBM tumors had remarkably higher mRNA and protein levels of FGL2, and the elevated FGL2 levels were shown to be related to a lower overall survival rate in GBM patients. By transferring delayed brain tumor glioma cells into mice, the authors constructed GBM murine models and found that anti-FGL2 antibody treatment led to significantly prolonged survival time. Moreover, the increased frequencies of CD4+CD39+Tregs, M2 macrophages, and myeloid-derived suppressor cells (MDSCs) were corrected after anti-FGL2 antibody treatment in GBM mice [100]. Further study by the same group identified that sFGL2 induced the co-inhibitory receptor TIGIT expression in brain-infiltrated T cells. Elevated TIGIT expression in T cells could suppress the anti-tumor immune responses in glioma, which might be a possible mechanism by which sFGL2 promoted tumor progression [101]. Additionally, Sun et al. reported that sFGL2 levels were considerably increased in hepatocellular carcinoma (HCC) patients. sFGL2 protein secreted by LX2 cells suppressed CD8+ T cell proliferation of HCC patients in a dose-dependent manner in vitro, and blockade of sFGL2 with antibody enhanced the proliferation of CD8+ T cells significantly [102]. These data indicate that FGL2 is a key immune-suppressive modulator in tumors and may serve as a new target for tumor immunotherapy.

### sFGL2 in other diseases

Aside from the above-mentioned diseases, roles of sFGL2 have been investigated in several other disorders. It has been reported that mRNA and protein levels of sFGL2 were significantly higher in mice experimentally infected with Echinococcus multilocularis [103]. The parasite loads, parasite proliferation activity, and proportion of parasite invaded liver were significantly lower in fgl2-/- mice compared to WT mice, suggesting the crucial roles of sFGL2 in Echinococcus multilocularis infection [104, 105]. Additionally, microarray analysis of lymphatic tissue from HIV infected patients revealed that FGL2 upregualtion might be related to the transition from acute to asymptomatic stage [25].

## CONCLUDING REMARKS

During the past few years, a flurry of emerging evidence has established the immunoregulatory role of sFGL2 as a novel effector molecule of Tregs, and considerable progress has been made in understanding the function of sFGL2 in normal and disease physiology. Nevertheless, much remains unclarified in regard to the upstream signals and precise downstream pathways that mediate the effects of sFGL2. Also, roles of sFGL2-related biological processes such as apoptosis in infection or transplantation immunology still need to be further defined. Collectively, further exploration of sFGL2 will deepen our understanding about the induction and maintenance of immune tolerance, thus facilitating the development of novel strategies for the management of multiple immune-related diseases, such as graft rejection, viral hepatitis and autoimmunity.
